# Vitamin C Attenuates Oxidative Stress, Inflammation, and Apoptosis Induced by Acute Hypoxia through the Nrf2/Keap1 Signaling Pathway in Gibel Carp (*Carassius gibelio*)

**DOI:** 10.3390/antiox11050935

**Published:** 2022-05-09

**Authors:** Liyun Wu, Wenjie Xu, Hongyan Li, Bo Dong, Hancheng Geng, Junyan Jin, Dong Han, Haokun Liu, Xiaoming Zhu, Yunxia Yang, Shouqi Xie

**Affiliations:** 1 State Key Laboratory of Freshwater Ecology and Biotechnology, Institute of Hydrobiology, Chinese Academy of Sciences, Wuhan 430072, China; wuliyun@ihb.ac.cn (L.W.); wenjie@ihb.ac.cn (W.X.); lihongyan@prfri.ac.cn (H.L.); dongbo@ihb.ac.cn (B.D.); genghancheng@ihb.ac.cn (H.G.); hand21cn@ihb.ac.cn (D.H.); liuhaokun@ihb.ac.cn (H.L.); xmzhu@ihb.ac.cn (X.Z.); yxyang@ihb.ac.cn (Y.Y.); sqxie@ihb.ac.cn (S.X.); 2 University of Chinese Academy of Sciences, Beijing 100049, China; 3 The Innovative Academy of Seed Design, Chinese Academy of Sciences, Beijing 100101, China

**Keywords:** hypoxia, vitamin C, Nrf2/Keap1 signaling pathway, oxidative stress, inflammation, apoptosis

## Abstract

Previous studies have found that vitamin C (VC) has protective effects in fish. However, the efficacy of VC on hypoxia-induced liver injury in fish remains unknown. Therefore, to investigate the protective mechanism of VC on liver injury after acute hypoxic stimulation in fish, gibel carp were fed a diet containing VC for eight weeks, then were subjected to acute hypoxia stimulation. The specific growth rate of fish was increased by the supplementation of VC. Plasma stress markers (glucose, lactic acid, and cortisol) were decreased by the VC supplementation. Moreover, the levels of the inflammatory cytokines (*tnf-α*, *il-2*, *il-6*, and *il-12*) were increased by enhancing the Nrf2/Keap1 signaling pathway. Upregulation of the antioxidant enzymes activity (CAT, SOD, and GPx); T-AOC; and anti-inflammatory factors (*il-4* and *tgf-β*) highlighted the antioxidant and anti-inflammatory activities of VC. The results showed that VC reduced the apoptotic index of the fish hypothalamus. The expression of GRP78 protein in the liver and endoplasmic reticulum stress and apoptosis induced by hypoxia were inhibited by VC. Taken together, the results indicate that VC can attenuate oxidative damage, inflammation, and acute hypoxia induced apoptosis in gibel carp via the Nrf2/Keap1 signaling pathway. The results identify a new defense strategy of gibel carp in response to hypoxic conditions.

## 1. Introduction

In mammals, oxygen is critical for survival, and it is an indispensable substrate for cell metabolism, energy balance, and signal transmission. Hypoxia-inducible factor-1α (HIF-1α), a major regulator of oxygen homeostasis, is highly expressed under hypoxic conditions [1]. Hypoxic conditions trigger the sharp increase of reactive oxygen species and oxidative stress, resulting in protein, DNA, and lipid damage, mitochondrial dysfunction, and apoptosis [2]. HIF can mediate the expression of inflammatory cytokines in brain and renal ischemic diseases, an important factor in resisting inflammatory infection [3,4].

Reactive oxygen species (ROS) formation can be affected by changes in oxygen concentration, causing oxidative stress when the levels exceed the removal ability of defense mechanisms [5]. ROS have been shown to regulate the active expression of HIF [6]. The liver is the main site of ROS production and the primary target of injury. ROS are critical in signal transduction, but excess ROS can trigger oxidative stress and disrupt intracellular homeostasis. Increased or decreased activity of antioxidant defense enzymes, such as superoxide dismutase (SOD), catalase (CAT), and glutathione peroxidase (GPx), can be used to maintain organism balance and reduce cell damage [7,8]. As one of the most important cellular defense mechanisms, nuclear factor erythroid 2-related factor (Nrf2) is a key transcription factor regulating the expression of genes involved in the antioxidant and anti-inflammatory responses. The key pathway of cellular resistance to oxidative stress was found to be Nrf2 (nuclear factor erythroid 2-related factor 2)/Keap1 (kelch-like ECH-associated protein 1 [9]. The Nrf2/Keap1 pathway has detoxification and neutralization effects, and many antioxidant enzymes regulated by the Nrf2/Keap1 pathway can remove reactive oxygen species; this not only resists oxidative damage from the external environment but also enhances the antioxidant capacity of the body [10]. It has been reported that resveratrol alleviates endoplasmic reticulum (ER) stress and apoptosis in brain tissue induced by explosion injury in mice through the Nrf2/Keap1 pathway [11]. In lipopolysaccharide-induced acute lung injury in mice, hispolon inhibited ER stress-mediated apoptosis and autophagy by regulating the Nrf2/Keap1 pathway [12]. In addition, studies on Wuchang bream (*Megalobrama amblycephala*) and gibel carp (*Carassius gibelio*) showed that emodin can mediate the Nrf2/Keap1 signaling pathway to improve the body’s antioxidant capacity and protect it from oxidative stress [13,14]. In recent years, a large amount of evidence has demonstrated that the Nrf2/Keap1 pathway is activated in various cellular and animal models of oxidative stress injury and is involved in tissue homeostasis and inflammatory responses [15].

An anoxic environment is closely related to the ER stress response. The ER has the structural and functional characteristics of organelles that can trigger stress responses and activate the unfolded protein (UPR) signaling pathway under hypoxia. It has been reported that the UPR primarily protects against oxidative stress damage caused by hypoxia through triggering autophagy. This mechanism attempts to rescue cells from damage from ER stress, and when this pathway is absent or disabled, cells are more sensitive to hypoxia-induced death [16]. In human cancer cells, severe hypoxia and ER stress trigger the transcription of autophagy-related genes by activating transcription factor 4 (ATF4) expression, and this upregulation is crucial for hypoxia and metabolic stress in tumors [17].

As an essential micronutrient and powerful antioxidant, vitamin C (VC) has been shown to have multiple biological properties that regulate antioxidant, antiaging, and anti-inflammatory activities and mediate cell death pathways [18]. Previous studies have shown that VC protects against chemically induced liver damage in mice [19]. In addition, VC deficiency can increase the levels of proinflammatory factors in grass carp and reduce the levels of anti-inflammatory factors, such as interleukin (IL) 10 and transforming growth factor (TGF) 1β. In addition, VC also promoted the expression of caspase-3, caspase-7, and caspase-9 and aggravated the apoptosis of grass carp (*Ctenopharyngodon idella*) cells [20]. It has been reported that VC can improve the antioxidant, antiapoptotic, and immune abilities of the abalone *Haliotis discus hannai* Ino [21].

When aquatic animals are starved of oxygen, they exhibit metabolic disorders and oxidative damage, ultimately leading to death [22]. Therefore, reducing tissue oxidative damage caused by hypoxia is the key to improving the survival of aquatic animals. Gibel carp (*Carassius gibelio*) is an economically important freshwater fish in China. Gibel carp may experience hypoxia in intensive culture; this not only harms the ecosystem and affects the diversity of aquatic resources, but it also causes large-scale asphyxia death of cultured fish. To date, there are no reports concerning whether VC treatment can alleviate acute hypoxic stimulation in gibel carp. In addition, the relationship between VC and the Nrf2/Keap1 pathway in response to acute hypoxia in gibel carp remains unknown. The purpose of this study was to explore whether dietary VC could alleviate acute hypoxia stress suffered by gibel carp. To this end, we performed a systematic study of the response of gibel carp to hypoxia from the aspects of immune response, endoplasmic reticulum stress, autophagy, and apoptosis, and the regulation of the Nrf2/Keap1 pathway in order to provide effective strategies for alleviating the fish response to hypoxic stress.

## 2. Materials and Methods

### 2.1. Ethical Statement

Procedures related to animal treatment in this study were approved by the ethics committee of the Institute of Hydrobiology, Chinese Academy of Sciences. All of the experiments were strictly in accordance with the Guidelines for the Management and Use of Laboratory Animals.

### 2.2. Experimental Diets

White fishmeal, rapeseed meal, and soybean meal were used as the main protein sources, and fish oil and soybean oil (1:1) were used as lipid sources to prepare two isonitrogenous and isolipic experimental diets; 1200 mg/kg VC was added to the basal diet (CON). VC (purity: 35%) was purchased from Guangzhou Liankun Biotechnology Co., Ltd. (Guangdong, China) and was added to the formula in the form of L-ascorbyl-2 monophosphate. The formulation and basic chemical composition of the diets are listed in Table 1. All of the ingredients were crushed through a 40-mesh sieve and thoroughly mixed according to the relevant formula ratio. Finally, oil was added and mixed. After mixing with water, a granulating machine (SLR-45, Fishery Machinery and Instrument Research Institute, Chinese Academy of Fishery Sciences, Shanghai, China) was used for granulating the feed, and the feed was then stored in a refrigerator at 4 °C.

### 2.3. Experimental Fish and Feeding Management

All of the gibel carp used in this experiment were provided by the Institute of Hydrobiology, Chinese Academy of Sciences (Wuhan, Hubei). Before the formal experiment, the experimental fish were temporarily raised in a tank and fed a temporary diet (36% crude protein and 7% crude lipid) for 14 days to adapt to the experimental conditions. Before the start of the experiment, the experimental fish were removed after being starved for 24 h, and healthy fish with similar specifications (6.66 ± 0.01 g) were randomly assigned to indoor fiberglass tanks for the experiment, with three tanks for each treatment and 30 fish in each tank. During the experiment, the experimental fish were fed to apparent satiety at 8:30, 13:30 and 18:30. The water temperature was measured every day; the fluctuation range was 29–31 °C. Dissolved oxygen, ammonia nitrogen, and pH were monitored weekly, with dissolved oxygen >6.0 mg/L, total ammonia nitrogen <0.1 mg/L, and pH 7.0–7.4. The aquaculture experiment lasted for 56 days.

### 2.4. Hypoxia Stress Test

After the eight-week feeding period, fish were randomly assigned to a CON group (control group), VC group (vitamin C group), HYPO group (hypoxia group), or HYPO + VC group (hypoxia + vitamin C group). During the hypoxia stress experiment, six healthy and uniform gibel carp were selected from each treatment and placed in a 1-L transparent plastic tank. The oxygen concentration of the hypoxia workstation (Ruskinn INVIVO_2_ 400, Beijing Longfujia Biotechnology Co., Ltd, Beijing, China) was adjusted to 2% to create a closed hypoxic environment, and the fish were moved into the hypoxia workstation for close monitoring. Before the stress test began, the oxygen concentration (6.14 ± 0.09 mg/L) in the fish tank was measured with an LDO101 probe (HQ30D, HACH). After being stressed in the hypoxic incubator for 3 h, the oxygen concentration in the cylinder was measured again; the oxygen concentration at this point was 0.08 ± 0.02 mg/L.

### 2.5. Sampling Procedures

After the feeding period, the fish were starved for 24 h, and the contents of the digestive tract were emptied. Then, all of the fish in each tank were weighed. Two fish with similar measurements were selected from each tank to be wiped dry, weighed, and stored at −20 °C for body composition analysis. Then, two fish were randomly chosen from each cylinder and put into a hypoxia incubator for three hours. Blood was taken from the caudal vein using a syringe soaked with heparin sodium (concentration: 0.2%), put into an anticoagulation centrifuge tube, and centrifuged (3000× *g* at 4 °C) for 15 min. The supernatant plasma was taken and stored in a refrigerator at −80 °C for the determination of plasma glucose, lactic acid, and cortisol. After the blood was drawn, the fish was dissected, and the liver tissues samples were taken, wrapped in sterilized tin foil paper, and immediately put into liquid nitrogen for subsequent analyses. In addition, brain tissue (hypothalamus) was collected and promptly placed in 4% paraformaldehyde fixative for histological analysis.

### 2.6. Biochemical Parameters

In this experiment, the determination of basic composition of all of the feed and fish body end samples was strictly in accordance with the AOAC (2003) standard methods [23]. The specific operation process was carried out according to Li et al. (2019) [24]. The moisture content was determined by the weight loss method. The ash content was determined after calcination in a muffle furnace (Hubei, China) at 550 °C for 3 h. Crude protein was determined by the Kjeldahl method (FOSS Tecator, Haganas, Sweden). Crude lipid was extracted and determined by a Soxtec system (Soxtec System HT6, Tecator, Haganas, Sweden). Gross energy was measured using an oxygen bomb calorimeter (Calorimeter, Parr instrument Company, Moline, IL, USA).

The liver enzyme activity needed to be measured before the experimental treatment. Physiological saline was added to liver samples according to the ratio of weight (g): volume (mL) = 1:9. After centrifugation, the supernatant was removed for later enzyme activity analysis. Protein concentration in a liver homogenate was determined by the Coomassie bright blue method (Bradford, P0006, Beyotime, Shanghai, China). Plasma lactic acid (LD) and total anti-oxidant capacity (T-AOC), superoxide dismutase (SOD), catalase (CAT), reduced glutathione (GSH), glutathione peroxidase (GPX), and the concentration of malondialdehyde (MDA) were determined using commercial kits (Nanjing Jiancheng Bioengineering Institute, Catalog: A019-2-1, A015-2, A001-3-2, A007-1-1, A006-2-1, and A005-1-2 and A003-1-2). The activities of Caspase 3 (C1116) and Caspase 9 (C1158) in the liver were detected using commercial kits (Beytime Biotechnology, Shanghai, China). The content of plasma cortisol was detected by enzyme linked immunosorbent assay (ELISA) (H094) provided by the Nanjing Jiancheng Bioengineering Research Institute. The principle of the method is to detect the content of cortisol in the sample by competition, i.e., adding the sample to the enzyme-labeled wells containing antibodies, then adding biotin-labeled recognition antigens that competed with the solid antibodies to form an immune complex, then adding avidin-HRP after washing, thus producing a yellow color under the action of acid with an absorption peak at a wavelength of 450 nm. Plasma glucose was determined using commercial kits (Fujifilm, Wako Pure Chemical Corporation, Osaka, Japan). The plasma samples were placed on ice, and a 96-well microenzyme assay was used. The plasma samples of 2 µL and different standard liquid concentrations were added into the 300-µL reaction mixture and mixed evenly. The plasma samples were placed in an incubator at 37 °C for 5 min away from light, and then the absorbance was read at 505 nm with a microenzyme reader.

### 2.7. Tissue Total RNA Extraction and Real-Time Fluorescence Quantitative PCR

In this experiment, the mRNA expression level of the liver tissue was analyzed, and the total RNA was extracted using the Trizol reagent (Invitrogen, Carlsbad, CA, USA). The detection of total RNA integrity primarily relied on 1.0% agarose electrophoresis, and then, 1 μL was taken to detect the purity and concentration of the sample by NanoDrop^®^ND-2000 ultramicro spectrophotometry (NanoDrop Technologies, Wilmington, DE, USA). The reverse transcription was performed using an M-MLV First-Strand Synthesis System according to the manufacturer’s instructions. Real-time fluorescence quantification was performed on a LightCycle 480 II (Roche Diagnostics, Basel, Switzerland) instrument to determine the amplification efficiency in a pre-experiment and to make standard curves using internal reference and target genes. SYBR Green I Master Mix (Roche Diagnostics, Indianapolis, IN, USA) fluorescent staining was used to determine the expression levels of all of the target genes. The calculation method of the relative expression refers to Pfaffl [25]. In this study, three housekeeping genes were employed initially: *β-actin*, *tubulin*, and *ef-1α*. After the analysis, it was determined that *ef-1α* had the highest stability, and thus, it was selected as a housekeeping gene. The target gene primers used in the experiment refer to a previous study [14], and the other primers are listed in Table 2.

### 2.8. TUNEL Staining

Fresh brain tissue (hypothalamus) was immobilized in 4% paraformaldehyde solution and then made into paraffin sections. The sections were repaired and permeated with protease K (2 mg/mL) and Trition-X-100/PBS (0.2%) solutions, and the TUNEL reagent was mixed with appropriate reagents 1 (TdT) and 2 (dUTP) at a ratio of 2:29 according to the tissue size, then incubated at 37 °C for 2 h. Slices were washed with PBS three times, each time for 5 min, and then DAPI dye solution (4′,6-diamidino-2-phenylindole, 0.3 mmol/L) was added and incubated at room temperature in the dark for 10 min. The sections were examined under a fluorescence microscope, and images were collected. Three fields were randomly selected for each section to be photographed. Image-Pro Plus 4.1 software was used to count and analyze the apoptosis rate calculated as follows:

Apoptosis rate (%) = The number of apoptotic nuclei/The number of observed nuclei × 100%.

### 2.9. Western Blot Analysis

The protein levels of HIF-1α, Nrf2, Keap1, and BiP/GRP78 in the liver tissue after hypoxic stress were detected by Western blotting. The liver samples were weighed and put into 1.5-mL centrifuge tubes, and protein lysate was added in proportion. The lysate consisted of a protease inhibitor, phosphatase inhibitor (Roche, Basel, Switzerland), and RIPA cleavage buffer (Beyotime Biotechnology, Shanghai, China). Then, ultrasonic crushing homogenization was carried out on ice, followed by centrifugation at 4 °C for 10 min at 13,000× *g*, and the supernatant was taken for later use. The sample protein concentration was measured according to the instructions of a BCA protein quantitative kit (Beyotime Biotechnology, Shanghai, China), and 50% RIPA lysate was used to adjust the protein concentration of all of the liver tissue samples to be consistent. The protein samples were then separated and transferred to polyvinylidene fluoride (PVDF) membranes after sodium dodecyl sulfate-polyacrylamide gel electrophoresis (SDS-PAGE). TBST was prepared with 5% skim milk powder at room temperature, and the PVDF membranes were sealed for 1 h. The specific primary antibody was then incubated at 4 °C for 12 h, followed by shaking cleaning with a TBST room temperature shaker three times for 20 min each time. Specific antibodies HIF-1α, Nrf2, BiP/GRP78, and GAPDH refer to a previous study [14]. Keap1 antibody (ab119403, Abcam) was incubated with a secondary antibody at room temperature for 2 h. The signal intensity was measured using an imager, and protein bands were obtained. The optical density of the protein bands was quantified using ImageJ software (National Institutes of Health, Bethesda, MD, USA). All of the values were normalized to the internal control (GAPDH) values.

### 2.10. Statistical Analysis

All of the data were verified for normality and homogeneity of variance before analysis using SPSS (SPSS Inc., Chicago, IL, USA) software. Data were expressed as the mean ± SE (standard error), and *p*-values < 0.05 or <0.01 were considered to be significant and extremely significant differences, respectively. The one-way ANOVA used in this experiment targeted the CON and the HYPO groups, as well as the HYPO + VC group.

## 3. Results

### 3.1. Growth Results and Body Composition

The results of the growth performance of gibel carp are shown in Table 3. The final body weight and specific growth rate of gibel carp fed the VC diet were significantly higher than those of the control group after eight weeks. Compared with the control group, no significant differences in feed utilization rate or feed efficiency were found in the VC group. Similarly, no significant differences were found in the crude protein, crude lipid, ash, or moisture content between the control group and the VC group.

### 3.2. Plasma Metabolites

**Figure 1:**
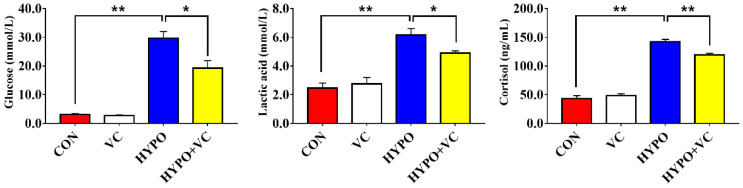
Plasma metabolites of gibel carp in the control and normal groups and hypoxia groups after feeding with VC for 56 days. The * or ** at the top of the bar chart indicates significant differences between treatments; * indicates a significant difference (*p* < 0.05), and ** indicates an extremely significant difference (*p* < 0.01). All of the data are presented as the mean ± standard error (n = 6).

The plasma metabolite levels of gibel carp after acute hypoxia stimulation are shown in Figure 1. The plasma glucose, lactic acid, and cortisol levels were increased significantly by acute hypoxia. However, all of the levels were significantly decreased in the VC-supplemented group.

### 3.3. Changes of HIF-1α Protein in the Liver

**Figure 2:**
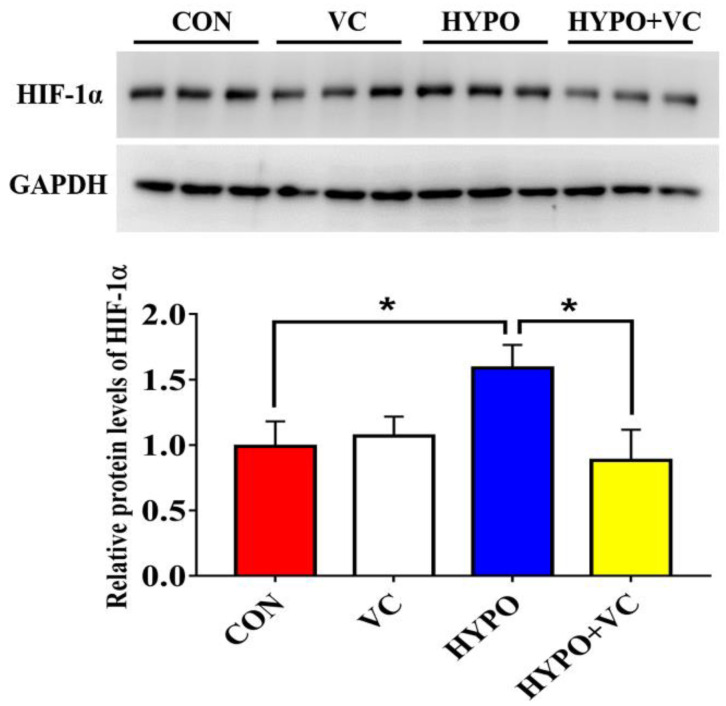
Changes in HIF-1α protein expression in the liver of gibel carp in normal and hypoxia groups after 56 days of feeding with VC. The * at the top of the bar chart indicates significant differences between treatments, * indicates a significant difference (*p* < 0.05). All of the data are presented as the mean ± standard error (n = 6).

Expression of the HIF-1α protein in the liver tissue is illustrated in Figure 2. The expression of HIF-1α protein in the liver tissue of gibel carp increased significantly after the hypoxia treatment. However, the HIF-1α levels were significantly decreased after VC supplementation.

### 3.4. Expression of Nrf2/Keap1 Signaling Pathway and Antioxidant-Related Genes in the Liver

**Figure 3:**
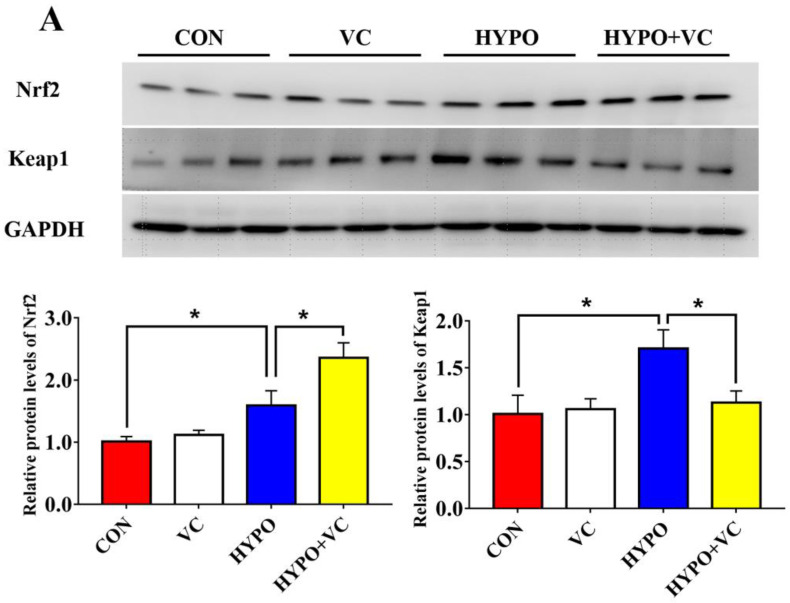

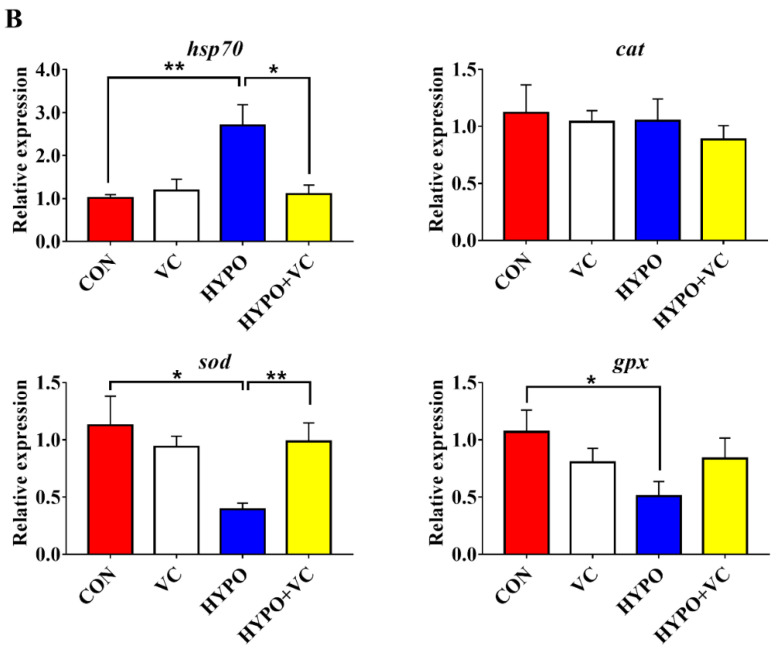
After 56 days of diet supplemented with VC, the expression levels of Nrf2 and Keap1 protein (**A**) and the expression of related genes (**B**) involved in antioxidant the Nrf2/Keap1 pathway in the liver of gibel carp were altered between the normal and hypoxia groups. The * or ** at the top of the bar chart indicate significant differences between treatments; * indicates a significant difference (*p* < 0.05), and ** indicates an extremely significant difference (*p* < 0.01). All of the data are presented as the mean ± standard error (n = 6). *hsp70—*heat shock protein 70; *cat—*catalase; *sod—*superoxide dismutase; *gpx—*glutathione peroxidase.

As shown in Figure 3, the protein levels of Nrf2 and Keap1 increased significantly in the liver after acute hypoxia stimulation. Moreover, acute hypoxia induced a significant increase in the expression level of *hsp70* in the liver and significantly decreased the expression of antioxidant-related genes, such as *sod* and *gpx*, while there was no significant effect on the expression of *cat*. Meanwhile, in the HYPO + VC group, Nrf2 protein expression was further activated, and Keap1 protein expression was decreased. In terms of genes, VC supplementation significantly reduced the expression of *hsp70* and increased the expression of *sod*, but it had no significant effect on the expression of *gpx* or *cat*.

### 3.5. Antioxidant Enzyme Parameters in the Liver

**Figure 4:**
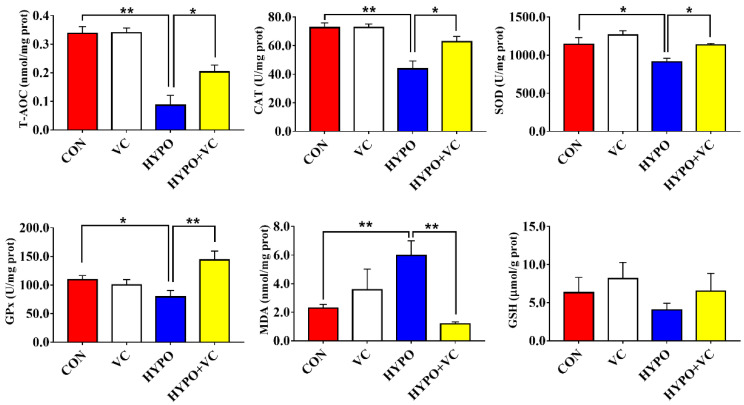
Changes in antioxidant enzyme parameters in the liver of gibel carp in the normal and hypoxia groups after 56 days of feeding with VC. The * or ** at the top of the bar chart indicates a significant difference between treatments; * indicates a significant difference (*p* < 0.05), and ** indicates an extremely significant difference (*p* < 0.01). All of the data are presented as mean ± standard error (n = 6). T-AOC*—*Total antioxidant capacity; CAT*—*Catalase; SOD*—*Superoxide dismutase; GP*—*Glutathione peroxidase; MDA*—*Malondialdehyde; GSH*—*Reduced glutathione.

The results of the antioxidant enzyme parameters in the liver are shown in Figure 4. The activities of the antioxidant enzymes CAT, SOD, and GPx in fish liver were significantly decreased after acute hypoxia treatment. T-AOC was also decreased, while the content of MDA was significantly increased. However, the antioxidant parameters were significantly enhanced, while the MDA content was significantly reduced in the HYPO + VC group. However, no change in the GSH content was found in this group.

### 3.6. Expression of Inflammation-Related Genes in the Liver

**Figure 5:**
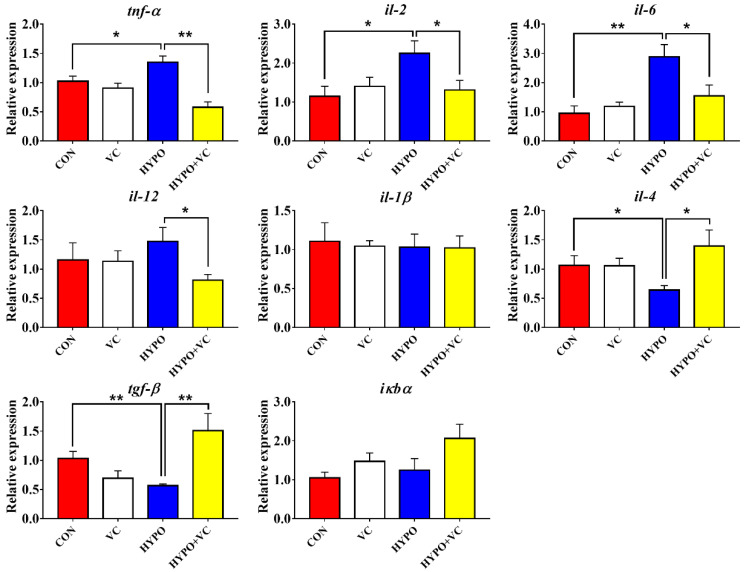
Changes of inflammation-related genes in the liver of gibel carp in the normal and hypoxia groups after 56 days of feeding with VC. The * or ** at the top of the bar chart indicates significant differences between treatments; * indicates a significant difference (*p* < 0.05), and ** indicates the extremely significant difference (*p* < 0.01). All of the data are presented as the mean ± standard error (n = 6). *tnf-α*—tumor necrosis factor-α; *il-2*—interleukin 2; *il-6*—interleukin 6; *il-12*—interleukin 12; *il-1β*—interleukin 1β; *tgf-β*—transforming growth factor-β; *il-4*—interleukin 4; *iκbα*—nf-κb inhibitorα.

Changes of the inflammation-related genes in the liver are shown in Figure 5. Gene expression levels of proinflammatory cytokines *tnf-α*, *il-2*, and *il-6* in gibel carp liver were significantly increased in the acute hypoxia group compared with the control group. The VC treatment significantly decreased the gene expression levels of *tnf-α*, *il-2*, *il-6*, and *il-12*. The transcript levels of anti-inflammatory cytokines *il-4* and *tgf-β* were inhibited by acute hypoxia. The gene expression levels of *il-4* and *tgf-β* were significantly increased in the VC-fed group after acute hypoxic stress. The mRNA levels of *il-1β* and *iкbα* were unaltered after the hypoxia treatment in all of the groups.

### 3.7. Expression of ER Stress Key Proteins and Related Genes in the Liver

**Figure 6:**
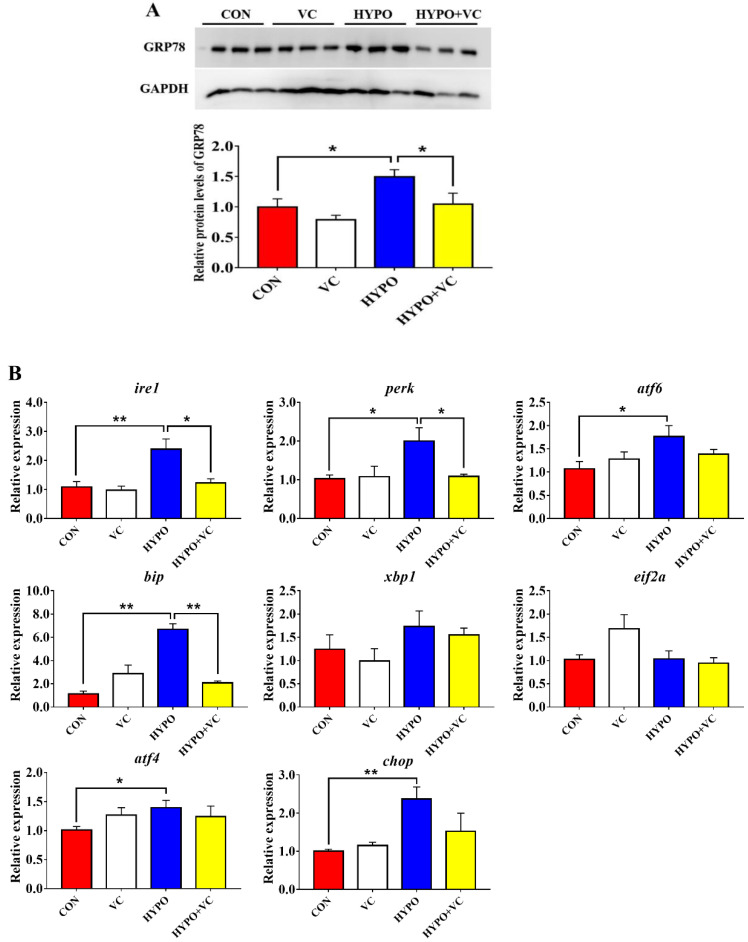
Changes in the expression levels of endoplasmic reticulum stress key protein, (**A**) and related genes (**B**) in the liver of gibel carp in normal and hypoxia groups after 56 days of feeding with VC. The * or ** at the top of the bar chart indicates significant differences between treatments; * indicates a significant difference (*p* < 0.05), and ** indicates an extremely significant difference (*p* < 0.01). All of the data are presented as the mean ± standard error (n = 6). GRP78—glucose-regulated protein 78; *ire1*—inositol-requiring protein-1a; *perk*—eukaryotic translation initiation factor 2-alpha kinase 3; *atf6*—activating transcription factor 6; *bip*—binding immunoglobulin protein; *xbp1*—X-box-binding protein 1; *ei2fa*—eukaryotic translation initiation factor 2A; *atf4*—activating transcription factor 4; *chop*—DNA damage-inducible transcript 3 protein.

As shown in Figure 6A, the expression levels of GRP78 protein in gibel carp liver were significantly increased after acute hypoxia stimulation. However, the GRP78 protein levels were significantly decreased in fish fed the VC diet. The transcript levels of *ire1*, *perk*, *atf6, bip*, atf6, and *chop* were significantly higher in the hypoxia group compared with the control group (Figure 6B). However, *xbp1* and *eif2a* mRNA levels were not affected by hypoxic stress. In the HYPO + VC group, the expression of the ER stress-related GRP78 protein and *ire1*, *perk*, and *bip* genes was significantly decreased. There were no significant differences in *atf4*, *atf6*, or *chop* mRNA levels. In addition, the gene expression levels of *xbp1* and *eif2a* were unaltered among all of the treatments.

### 3.8. Expression of Autophagy and Apoptosis-Related Genes in the Liver

**Figure 7:**
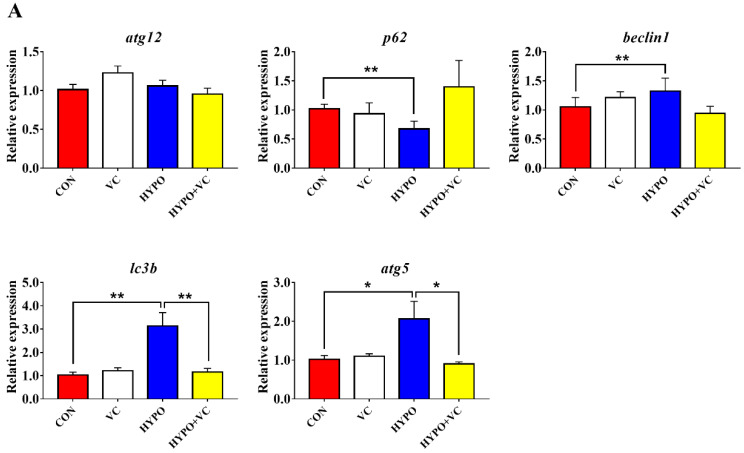

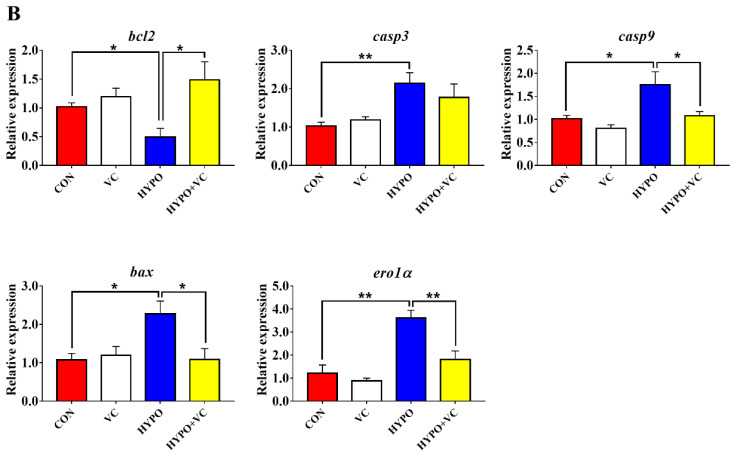
Changes of the expression levels of autophagy (**A**) and apoptosis (**B**)-related genes in the liver of gibel carp in the normal group and hypoxia group after 56 days of feeding with VC. The * or ** at the top of the bar chart indicates significant difference between treatments; * indicates a significant difference (*p* < 0.05), and ** indicates an extremely significant difference (*p* < 0.01). All of the data are presented as the mean ± standard error (n = 6).

The changes of autophagy-related genes, including *atg12*, *p62*, *beclin1*, *lc3b*, and *atg5*, in gibel carp liver after acute hypoxia stimulation are presented in Figure 7A. In the liver, the expression levels of *beclin1*, l*c3b*, and *atg5* were significantly increased after the acute hypoxia stimulation compared with the control group. The opposite change was found in the *p62* mRNA levels. Dietary supplementation with VC significantly inhibited the gene expression levels of *lc3b* and *atg5*, while there were no significant differences in the gene expression levels of *p62* or *beclin1*. In addition, the expression levels of *atg12* were unchanged, irrespective of treatment.

The relative expression of apoptosis-related genes in the liver tissue is shown in Figure 7B. The gene expression of *bcl2* was decreased significantly after acute hypoxia. The expression levels of proapoptotic genes *bax* and *casp3*, *casp9* and *ero1α* were significantly induced by hypoxia. However, the expression levels of *bcl2* and *casp9* were significantly increased after VC supplementation, while the *bax* and *ero1α* transcript levels were significantly decreased.

### 3.9. Determination of Casp 3 and 9 Activities in the Liver

**Figure 8:**
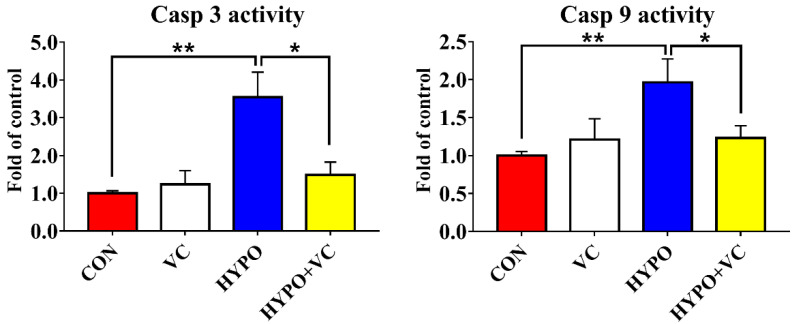
Changes of Casp 3 and Casp 9 activities in the livers of gibel carp in normal group and hypoxia group after 56 days of feeding with VC. The * or ** at the top of the bar chart indicates significant differences between treatments; * indicates a significant difference (*p* < 0.05), and ** indicates an extremely significant difference (*p* < 0.01). All of the data are presented as the mean ± standard error (n = 6).

Activities of Casp 3 and Casp 9 in the liver are shown in Figure 8. Acute hypoxia induced the activities of Casp 3 and Casp 9 compared with the control group. However, VC treatment significantly decreased the activities of Casp 3 and Casp 9 in the liver of gibel carp.

### 3.10. TUNEL Observations

**Figure 9:**
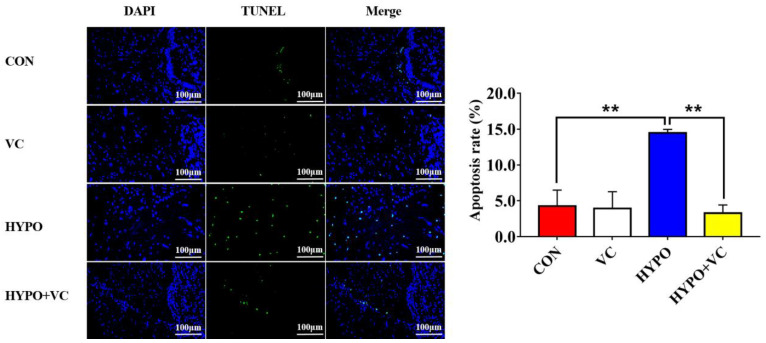
Changes of DAPI and TUNEL staining from representative sections in the hypothalamus of gibel carp in the normal group and hypoxia group after 56 days of feeding with VC. Positive apoptotic nuclei in green and normal nuclei are shown in blue. The magnification and scale of the microscope were 200× and 100 μm, respectively. The ** at the top of the bar chart indicates significant differences between treatments; ** indicates an extremely significant difference (*p* < 0.01). All of the data are presented as the mean ± standard error (n = 6).

The results of TUNEL and DAPI staining in the hypothalamus are presented in Figure 9. Apoptosis signals were increased significantly after acute hypoxia stimulation, and these were decreased in fish fed with VC diet after hypoxia.

## 4. Discussion

Vitamin C is extremely critical to maintaining normal growth and the antistress ability of fish. VC participates in a variety of physiological processes in the body, such as growth, development, and stress response, and it can protect the important functions of the immune system from oxidative damage. High doses of VC significantly improved the growth performance of Asian catfish (*Clarias batrachus*) and Wuchang bream (*Megalobrama amblycephala*) [26,27]. In line with this, a higher specific growth rate was found in fish fed the VC diet, indicating that VC improves the growth of gibel carp. However, no effect of supplementation with VC was found on the growth of large yellow croaker (*Pseudosciaena crocea*) [28]. The differences in the effects of VC may be related to the fish species and supplemental VC concentration.

As an energy substance metabolized by various tissues, the concentration of glucose in the blood is essential to maintaining life activities of fish. Normally, glycemia can be induced by stress in fish [29]. Consistent with this, glycemia was induced by hypoxia in gibel carp. As a metabolite, lactate content increases rapidly under hypoxic conditions [30]. In biochemical tests, the cortisol level is used as a characteristic index of fish stress intensity. It is one of the hormones secreted by the adrenal gland and can enter the blood directly [31]. The levels of cortisol in the hypoxia group were significantly increased, suggesting that hypoxia could induce a severe stress response in gibel carp. However, in the treatment group fed the VC supplemented diet, the levels of glucose, lactate, and cortisol in the plasma were significantly decreased, indicating that VC alleviated the hypoxic stimulation of gibel carp.

Hypoxia inducible factors (HIFs) are the most important transcription factor family that can regulate a variety of genes in response to decreases of intracellular oxygen concentration. HIF-1α overexpression can inhibit the apoptosis of K562 cells induced by reactive oxygen species [32]. In the present study, HIF-1α protein expression in the liver was significantly upregulated after acute hypoxic stimulation. Interestingly, VC supplementation alleviated the induction of HIF-1α protein expression by hypoxia, indicating a decreased response of fish to hypoxic stress. Additionally, VC has antioxidant and anti-inflammatory activities [18]. CAT and SOD activities were significantly downregulated after 12 h of acute hypoxia in the liver of largemouth bass (*Micropterus salmoides*) [33]. In the present study, compared with the control group, the activities of CAT, SOD, and GPx in the liver of gibel carp in the hypoxia group were downregulated, together with decreased T-AOC, implying that hypoxia leads to an excessive accumulation of ROS and the body possible severe oxidative damage. In hypoxia/reoxygenated rats, SOD and GSH activities in the brain were significantly downregulated, and the MDA content was significantly upregulated after stress [34]. CAT and GPx activities in the brain were induced by hypoxia in common carp (*Cyprinus carpio*); however, no significant difference in CAT activity in the liver or muscle was found, and GPx activity was significantly downregulated after hypoxia treatment [35]. Thus, the response of antioxidant levels may differ among tissues. In addition, excessive oxidative stress can cause hepatocyte apoptosis or necrosis, thereby indirectly initiating liver injury [36]. In the present study, oxidative stress was effectively inhibited by VC, consistent with previous reports [37,38]. The increases of ROS and oxidative stress are positively correlated with hypoxia, both of which are considered to be the main etiological factors leading to body dysfunction and damage and are important parts of the series of events leading to proinflammatory mediators and cell apoptosis. Hypoxia causes a massive accumulation of ROS that directly or indirectly oxidizes or damages DNA, proteins, and lipids; induces the formation of lipid peroxidation; and damages cell membranes [39]. Lipid peroxidation was the most sensitive indicator respond to hypoxia in goldfish [35]. MDA was significantly upregulated by hypoxia in gibel carp. MDA concentration was increased in largemouth bass after 24 h of acute hypoxia exposure [33]. The MDA content in the liver tissue was downregulated in VC-fed fish, indicating that VC plays a potential protective role in alleviating acute hypoxia-induced oxidative stress.

Nrf2 is a common regulator of the antioxidant system and immune inflammatory responses. The protein dissociates from Keap1 into the nucleus and plays a key role in antioxidant capacity, anti-inflammation, and anti-apoptosis when oxidative stress occurs [40,41,42]. The antioxidant capacity was improved through Nrf2/Keap1 signaling by a VC-supplemented diet in abalone [21]. In the present study, the Nrf2/Keap1 pathway was activated by acute hypoxia, as indicated by increased protein levels of Nrf2. Since increased protein expression of Nrf2 and decreased protein expression of Keap1 were found in VC-supplemented group, this indicated that VC was involved in the Nrf2/Keap1 pathway regulation of hypoxia. Inflammation is an important immune defense mechanism of the body, a state of self-protection or damage repair initiated when tissues are subjected to environmental stress or bacterial infection. In macrophages, Nrf2 activation prevents the onset of inflammatory responses [43]. In the present study, the expression levels of pro-inflammatory genes *tnf-α*, *il-2*, and *il-6* were upregulated in the hypoxia group, while the expression levels of anti-inflammatory genes *il-4* and *tgf-β* were downregulated, suggesting that acute hypoxia stimulated an inflammatory response in gibel carp. Upregulated expression of *tnf-α*, *il-2*, *il-6*, and *il-12* and downregulated expression of anti-inflammatory genes *il-4* and *tgf-β* induced by VC suggested that VC attenuated the expression of proinflammatory factors [44]. Thus, oxidative stress, inflammation, and apoptosis were attenuated by VC via the Nrf2/Keap1 pathway in gibel carp.

Various endogenous and exogenous stresses can destroy protein homeostasis in organelles, leading to ER damage and activating the UPR response [45]. UPR activation is closely related to pathological processes, such as inflammation and autophagy [46,47,48]. Protein kinase RNA-activated-like ER kinase (PERK), inositol-requiring enzyme 1α (IRE1α), and activated transcription factor 6 (ATF6) act as ER-located protein stress markers that can coordinate the response to harmful accumulation of unfolded or misfolded proteins. In the present study, VC treatment reduced the expression of GRP78, a marker protein of ER stress induced by acute hypoxia. Similarly, chrysophanol treatment also reduced hypoxia/reoxygenation-induced GRP78 protein expression [49]. Molecular chaperone binding immunoglobulin (BiP) is recognized as one of the most sensitive markers of ER stress, as it facilitates tumor growth through a variety of mechanisms such as promoting the maturation and secretion of growth factors, inhibiting apoptosis, and stimulating angiogenesis. In mice, VC treatment reduced ER stress induced by perfluorooctane sulfonate and downregulated the expression of ATF6 and GRP78 proteins [44]. Herein, the expression levels of *ire1*, *perk*, *atf6*, *bip*, *atf4*, and *chop* in the liver were significantly upregulated after acute hypoxia, indicating that acute hypoxia stimulation induced the activation of hepatic ER stress in gibel carp. However, *ire1*, *perk*, and *bip* genes in the liver tissue were significantly downregulated by VC supplementation. Therefore, VC may exert its pharmacological protective effect in the liver by inhibiting ER stress in fish.

Autophagy is a process by which eukaryotic cells utilize lysosomes to degrade cytosolic proteins and damaged organelles under the regulation of autophagy-related genes. Hypoxia can induce autophagy, and autophagy has dual functions in tissue damage caused by hypoxia. Under hypoxia, autophagy or autophagy-related proteins induce apoptosis by activating Caspase or by reducing endogenous apoptosis inhibitors, ultimately aggravating cell injury [50]. Under severe hypoxia, the PERK pathway upregulates the expression of *lc3b* by activating the expression of *atf4* and *chop* [51]. In myocytes, hypoxia causes upregulation of *beclin1* expression that simultaneously prompts the conversion of LC3B-I to LC3B-II and is recruited to the autophagosome outer membrane [52]. In the process of autophagy, LC3B-II extends throughout the autophagic process and is a currently recognized marker of autophagy. In the present study, *beclin1*, *lc3b*, and *atg5* mRNA levels were significantly upregulated in the hypoxia group, indicating that the autophagy process was initiated. However, the gene expression levels of *lc3b* and *atg5* were significantly downregulated in the VC-diet-fed group, implying that VC attenuated autophagic process induced by hypoxia in gibel carp. Severe autophagy induces apoptosis, and caspase is an essential part of the apoptosis mechanism that can reflect whether apoptosis occurs in the body [53]. Bax and Bcl2 play regulatory roles in apoptosis; both belong to the Bcl family, and they ultimately mediate apoptosis by controlling the permeability of mitochondria and sequentially activating caspase 9 and caspase 3 [54,55]. In the present study, apoptosis was activated by hypoxia, as indicated by increased expression levels of *bax* and *ero1α*. Increased expression levels of *bcl2*, concurrent with inhibited activities of Casp3 and Casp9 together with downregulated *casp9* expression induced by VC indicated that VC inhibited apoptosis induced by hypoxia in gibel carp, consistent with results in abalone [21]. Additionally, TUNEL-positive cells were significantly reduced in gibel carp fed a VC diet under hypoxia compared with fish in the control group, verifying the anti-apoptosis effects of VC. Taken together, the results suggest that VC enhanced the anti-apoptotic ability of gibel carp through inhibiting ER stress and caspase-dependent pathways.

## 5. Conclusions

Acute hypoxia stimulates oxidative damage, inflammation, and ER stress, resulting in apoptosis in gibel carp. VC protected fish from acute hypoxia injury by enhancing Nrf2/Keap1 signaling and downregulating the level of HIF-1α protein, thereby leading to inhibition of oxidative stress, inflammation, and apoptosis. This study provides new evidence for the hypothesis that VC supplementation can effectively alleviate oxidative stress and inflammation induced by acute hypoxia in fish.

## Figures and Tables

**Table 1:** Ingredients and proximate composition of the experimental diets (% dry matter).

Ingredients	CON	VC
White fish meal ^1^	15	15
Rapeseed meal ^2^	20	20
Soybean meal ^2^	25	25
Wheat flour	25.6	25.6
Oil mixture ^3^	5.5	5.5
Vitamin C	0	0.12
Vitamin premix ^4^	0.39	0.39
Choline chloride	0.11	0.11
Mineral premix ^5^	5	5
Carboxy methyl cellulose sodium	3	3
Cellulose	0.40	0.28
**Proximate analysis (dry matter)**		
Crude protein (%)	37.21	37.42
Crude lipid (%)	6.77	6.63
Moisture (%)	9.08	9.17
Ash (%)	10.02	10.34
Gross energy (kJ g^−1^)	19.27	19.22

^1^ White fish meal: Purchased from American Seafood Company, Seattle, WA, USA. ^2^ Soybean and rapeseed meal: Purchased from Coland Feed Co. Ltd., Wuhan, Hubei, China. ^3^ Oil mixture: soybean oil: fish oil = 1:1. ^4^ Vitamin premix (mg kg^−1^ diet): Vitamin B1, 20; Vitamin B2, 20; Vitamin B6, 20; Vitamin B12, 0.02; folic acid, 5; calcium pantothenate, 50; inositol, 100; niacin, 100; biotin, 0.1; cellulose, 3522; Vitamin C, 100; Vitamin A, 110; Vitamin D, 20; Vitamin E, 50; Vitamin K, 10. ^5^ Mineral salt premix (mg kg^−1^ diet): NaCl, 500.0; MgSO_4_·7H_2_O, 8155.6; NaH_2_PO_4_·2H_2_O, 12500.0; KH_2_PO_4_, 16000; Ca(H_2_PO_4_)·2H_2_O, 7650.6; FeSO_4_·7H_2_O, 2286.2; C_6_H_10_CaO_6_·5H_2_O, 1750.0; ZnSO_4_·7H_2_O, 178.0; MnSO_4_·H_2_O, 61.4; CuSO_4_·5H_2_O, 15.5; CoSO_4_·7H_2_O, 0.91; KI, 1.5; Na_2_SeO_3_, 0.60; Corn starch, 899.7.

**Table 2:** Sequences of primers applied for quantitative real-time PCR analysis in gibel carp.

Gene Name	Sense and Antisense Primer (5’–3’)	Gene Bank	Product Length
		Accession No.	(bp)
Tumor necrosis factor-α (*tnf-α*)	TTGAGCAGGAGATGGGAACCG	XM_026282152.1	115
	AGAGCCTCAGGGCAACGGAAA		
Interleukin-2 (*il-2*)	GACCACAAAGGTAGACCCATCC	MN338056	212
	GAGGTTTGTGCGGAATGGAC		
Interleukin-6 (*il-6*)	TGTTCTCAGGGCATTCGCTT	XM_026289280.1	161
	GGAGTTGTAGTGCCCTTGGT		
Interleukin-12 (*il-12*)	CTTCAGAAGCAGCTTTGTTGTTG	LN592213.1	77
	CAGTTTTTGAGAGCTCACCAATATC		
Interleukin-1β (*il-1β*)	TTTGTGAAGATGCGCTGCTC	AB757758.1	133
	CCAATCTCGACCTTCCTGGTG		
Interleukin-4 (*il-4*)	CGATTGTAGCCGTTACTGGGT	KX574595	166
	TGGCAAATGTGTTCCTCCG		
Transforming growth factorβ (*tgf-β*)	ATGAGGGTGGAGAGTTTAT	EU086521.1	155
	AGTCGTAGTTTGCTGAGAA		
Nuclear factor of kappa light polypeptide gene	TTGCGAATCCAAAGGGGACA	XM_026291433.1	196
enhancer in B-cells inhibitor, alpha (*iκbα*)	TCTGTGATGACGGCGAGATG		

**Table 3:** Effects of dietary VC on growth performance and body composition of gibel carp.

Category	Parameter	Group	
		CON	VC
**Growth performance**	Initial body weight (g)	6.60 ± 0.00	6.67 ± 0.03
	Final body weight (g)	26.40 ± 0.21 ^a^	31.40 ± 1.36 ^b^
	WGR ^1^ (%)	219.80 ± 12.98	261.38 ± 17.86
	FE ^2^ (%)	44.19 ± 0.42	50.79 ± 2.81
	SGR ^3^ (% d^−1^)	4.93 ± 0.03 ^a^	5.54 ± 0.16 ^b^
**Body composition**	Crude protein (%)	15.18 ± 0.16	14.91 ± 0.1
	Crude lipid (%)	7.35 ± 0.19	7.22 ± 0.04
	Ash (%)	2.68 ± 0.12	2.60 ± 0.13
	Moisture (%)	71.95 ± 0.54	71.81 ± 0.27

The data listed in the table are all expressed as the means ± SEM, and the superscripts (a or b) of different letters in the same row indicate significant differences (*p* < 0.05). ^1^ WGR: Feeding rate (%) = [(final weight (g) − initial weight (g))/initial weight (g)] × 100]. ^2^ FE: Feeding efficiency (%) = (100 × fresh body weight gain)/dry feed intake. ^3^ SGR: Specific growth rate (% d^−1^) = 100 × [ln (final weight) - ln (initial weight)]/day.

## Data Availability

Data are contained within the article.
